# Psychiatric Symptoms in Women with High-risk Pregnancy in the Postpartum Period: A Case-control Study

**DOI:** 10.1055/s-0043-1768997

**Published:** 2023-05-24

**Authors:** Zahra Basirat, Fatemeh Ramaezani, Mahdi Sepidarkish, Mahdi Kashifard, Mahbobeh Faramarzi

**Affiliations:** 1 Babol University of Medical Sciences, Babol, Iran

**Keywords:** Psychological disorders, High-risk pregnancy, Postpartum, Psychological distress

## Abstract

**Objective:** Psychiatric symptoms are common mental issues in pregnancy and the postpartum period. There is limited information regarding the psychiatric symptoms of women with high-risk pregnancy in the postpartum period. This study aimed to compare the severity of psychiatric symptoms and psychological distress in women with high-risk and low-risk pregnancies in the postpartum period.

**Methods:** This case-control study examined 250 women in the postpartum period in two groups with low-risk (n = 112) and high-risk (n = 138) pregnancies. Women completed the Brief Symptom Inventory-53 (BSI-53) and the Risk Postnatal Psychosocial Depression Risk Questionnaire (PPDRQ).

**Results:** The mean severity of psychiatric symptoms in women with high-risk pregnancies was significantly higher than that in women with low-risk pregnancies (39.34 ± 17.51 vs. 30.26 ± 17.08). Additionally, the frequency of psychological distress in women with high-risk pregnancies was approximately twice higher than that in women with low-risk pregnancies (30.3% vs. 15.2%). Furthermore, the risk factors for depression in women with high-risk pregnancies were almost 1.5 times (59.8% vs. 39.8%) higher than the factors in women with low-risk pregnancies. The results of the logistic analysis indicated that high-risk pregnancies could be twice the odds ratio of developing postpartum psychological distress (ß = 2.14, 95% CI 1.4-6.3, p= 0.036).

**Conclusion:** Psychiatric symptoms and the psychological distress index are higher in postpartum women with high-risk pregnancies than in postpartum women with low-risk pregnancies. The study suggests that obstetricians and pregnant women's health care providers should strongly consider screening of psychiatric symptoms in women with high-risk pregnancies both during pregnancy and after delivery as the women's routine care priorities.

## Introduction

Pregnancy and childbirth are important stages of a woman's life that increase the chance of mental disorders. 1 2 Numerous studies have confirmed a higher risk of psychiatric symptoms during the first months after delivery compared to the risk of the out-of-postpartum period. 3 Severe psychiatric disorders are events putting mothers and fetuses at risk for suicide and/or infanticide and are among developmental growth disorders for fetus or child. 4 5

Approximately 10 to 13% of women experience postpartum depression, 6 and among them, 20 to 60% also experience anxiety. 7 8 Delivery is an important life event accompanied by profound and usually stressful changes in all aspects of daily life, causing women to be vulnerable to psychiatric disorders. 9 The risk of severe postpartum psychological disorders may be related to hormone fluctuation rather than their concentration. 10

Although various risk factors have been identified for perinatal psychiatric illnesses, a review study indicates that medical complications during pregnancy are associated with postpartum psychiatric disorders. 11 The medical complications include preeclampsia, 3 12 hyperemesis gravidarum, 13 gestational diabetes, 14 15 gestational hypertension, 16 postpartum hemorrhage, 17 cesarean section, 18 and preterm delivery. 19 A meta-analysis study also reported preeclampsia as a risk factor for postpartum depression. 20

Further understanding of the relationship between high-risk pregnancy and postpartum psychiatric disorders is significant for the development of targeted screening and treatment approaches. Few studies have examined the range of psychiatric illnesses in the postpartum period, and most have focused on a disorder. To the best of our knowledge, the present research was the first case-control study comparing the severity of psychiatric symptoms to nine subscales and risk of postpartum depression in two groups of women with high-risk and low-risk pregnancies. Therefore, this study had three objectives: 1) comparison of the severity of nine symptoms of psychiatric disorders, and the psychological distress index in postpartum women with low-risk and high-risk pregnancies, 2) comparison of the scores of psychosocial risk factors in postpartum women with low-risk and high-risk pregnancies, 3) exploration of the determinants of psychological distress in women with psychological distress in the postpartum period.

## Methods

The present case-control study examined pregnant women who visited public or private midwifery clinics in Babol city from November 2020 to September 2021 to receive postpartum care. The initial inclusion criteria for both groups were as follows: age over 18 years, education level higher than primary school, vaginal delivery, the first 6 weeks after delivery, and consent to enter the study. Women, who experienced “traumatic birth” like an operative birth, large perennial lacerations, and infant malformation or death, were excluded from the study.

Inclusion criteria for the case group included having a high-risk pregnancy in a recent delivery so that the person should have at least one of the high-risk pregnancy criteria according to the pregnancy risk questionnaire. This questionnaire determines high-risk pregnancy based on several important factors, including demographic (age under 18, age over 35, low/high body mass index), history of medical diseases (cardiovascular, pulmonary, renal, and thyroid diseases), current status of high-risk pregnancy (gestational diabetes, hypertension, preterm delivery, fetal distress, and medical diseases, pregnancy following in vitro fertilization, and multiple pregnancy), and high-risk behaviors (substance abuse, alcohol, and smoking). 21

Inclusion criteria for the control group included low-risk pregnancies with no high-risk pregnancy symptoms. The participants in the low-risk pregnancy group were selected by matching them to the high-risk group. The matching method was based on the frequencies of age and education characteristics.

This study had a convenience sampling method. The sample size was obtained equal to 243 based on the clinical difference of 10 units in the score of the Brief Symptom Inventory using the sample size formula (considering the test power of 80%, α = 0.05, μ _1_ = 17, and μ _2_ = 12) with the PASS15 software.

One midwife outside the research team explained the purpose of the study to the patients. She interviewed the women during postpartum visits, while the women waited to visit a doctor. She examined the initial inclusion criteria by studying the medical record and obtaining a history from the patient. If the woman were eligible to include in the case group (high-risk) or control group (low-risk), she would tell her the research purpose. A high-risk pregnancy was considered when a person had one of the criteria for diagnosing a high-risk pregnancy according to the checklist. 21 If the person did not have any of the high-risk pregnancy criteria, she was assigned to the low-risk group. If the pregnant woman were willing to participate in the study, she would complete the informed consent form. Then, the women in both groups (112 with high-risk pregnancies and 138 with low-risk pregnancies) responded to the questionnaires of the study. The participants completed the following questionnaires:

**Brief Symptom Inventory-53 (BSI-53):** This questionnaire consists of 53 questions covering 9 domains of psychological symptoms, including depression (symptoms of mood swings and influences as well as lack of motivation and loss of interest in life), anxiety (restlessness and stress as well as panic attacks and feelings of fear), somatization (suffering from the perception of bodily dysfunction), obsessive-compulsive (thoughts and impulses experienced are incessant and irresistible but undesirable in nature), interpersonal sensitization personal (feeling of personal inferiority and inferiority to others), phobic anxiety (persistent fear response to a place, object, or situation that is not reasonable), paranoid ideas (disordered thinking characteristics of projective thoughts and hostility), hostility (thoughts, feelings, or actions characteristic of anger), and psychoticism (withdrawal, isolation, schizophrenia lifestyle as well as the leading symptoms of schizophrenia).

Psychological distress, Global Severity Index (GSI), was calculated with the sum of the scores of 53 questions divided by 53 questions. 22 The reliability of the Persian version of BSI-53 showed a Cronbach's alpha coefficient of 0.95 for all subscales. The cut-off point of the psychological distress index was obtained 1 based on the scores. 23

**Postnatal Psychosocial Depression Risk Questionnaire (PPDRQ** ): This questionnaire has 12 questions some of which are scored from 0 to 6. The score range of the questionnaire is from 8 to 82. Higher scores indicate a higher risk. 24 The Persian validity of the questionnaire was used in this study. The cut-off point of psychosocial risk was considered 23 based on the total scores. 25

The study data were statistically analyzed using SPSS 22. To compare the variables of the two groups, the independent t-test, chi-square test, and Fisher's exact test were used according to the type of variable and the assumptions of the tests. Furthermore, multivariate models were also used to control the effects of other confounding variables in the data analysis. Moreover, the Multiple Logistic Regression test was used. In this model, GSI > 1 was included as a dependent variable, while the psychosocial risk score ≥23, age, and education level were included as independent variables. The results were calculated and reported as an adjusted odds ratio with a confidence level of 95%. P values less than 0.05 were considered statistically significant.

## Results

The mean age of all women was 29.71 ± 5.42 years (minimum age of 17, and maximum age of 44). In terms of education level, 62 (24.8%) women had under-high-school-diploma degrees, 75 (30.0%) had high-school diplomas, and 113 (45.2%) had academic degrees and higher. Table 1 shows that the highest frequency belongs to the risk factor of age of women under 18 years or over 35 (26.8%) in women with high-risk pregnancies. Furthermore, the lowest frequency of pregnancy risk is related to renal diseases (1.1%).

**Table 1:** Frequency of maternal complications in women with high-risk pregnancy

Factors	n(%) (n = 112)
Maternal age of upper 35 year or	30 (26.9)
Hyperemesis gravid	10 (8.9)
Hypertension	7 (6.7)
Diabetes	16 (14.4)
Kidney disease	2 (1.1)
Treated abortion	9 (8.2)
Hemorrhage placenta previa or abruption	3 (2.8)
Pyelonephritis	10 (8.9)
Peterman labor	15 (13.5)
Intrauterine growth retardation	10 (8.9)

Table 2 compares the mean psychological symptoms with 9 subscales, the GSI, and the psychosocial risk score in the groups. The normal data distribution was measured using the Kolmogorov-Smirnov test. Based on the results, not all variables followed the normal distribution; hence, the nonparametric Mann-Whitney test was used. The results indicated that the mean scores of severe symptoms of obsession-compulsion, depression, anxiety, hostility, paranoid ideation, and psychoticism were significantly higher in postpartum women with high-risk pregnancies than in those with low-risk pregnancies (P < 0.05). Furthermore, the psychological distress index and high-risk factors were significantly higher in women with stillbirths and high-risk pregnancies than in those with low-risk pregnancies (P < 0.05). However, the two groups were not significantly different in terms of mean physical complaints, interpersonal sensitivity, and phobic anxiety. We also compared the frequencies of psychological distress and psychosocial risk in women with high-risk and low-risk pregnancies. According to the study of psychosocial risk based on the cut-off point 23, 59.8% (67.112) of women with high-risk pregnancies compared to 39.8% (55.138) of women with low-risk pregnancies had high psychosocial risk in terms of depression, and the difference was statistically significant (P= 0.04). The level of psychological distress (with GSI > 1) was 30.3% in women with high-risk pregnancies, and it was significantly higher than that in women with low-risk pregnancies (15.2%) (P= 0.004).

**Table 2:** The comparison of the mean of psychological symptoms in women with high-risk and low-risk pregnancy

	Low- risk pregnancy Mean (SD)	High-risk pregnancy Mean (SD)	P-value	Total population Mean (SD)
Somatization	5.16(3.99)	5.75(4.55)	0.283	5.42(4.25)
Obsession-computation	3.77(3.62)	4.66(4.51)	0.091	4.17(4.06)
Interpersonal Senility	2.71(2.84)	3.09(3.24)	0.326	2.88(3.03)
Depression	2.96(3.56)	4.27(5.08)	0.022	3.54(4.35)
Anxiety	3.77(3.95)	4.75(4.51)	0.072	4.21(4.23)
Hostility	2.17(2.19)	3.21(3.21)	0.004	2.64(2.75)
Phobic anxiety	1.63(2.26)	2.54(3.15)	0.011	2.04(2.73)
Paranoid ideation	3.73(3.65)	4.80(4.01)	0.028	4.21(3.84)
Psychoticism	1.83(2.53)	3.17(3.35)	0.001	2.43(3)
Total score BSI-53	30.17(26.08)	39.17(34.51)	0.022	24.14(9.29)
*GSI	0.56(0.49)	0.73(0.65)	0.022	26.42(9.29)
Risk of depression	25.57(8.38)	28.20(9.66)	0.022	0.54(0.57)

*GSI: Global Severity Index; BSI-53: Brief Symptom Inventory-53.

Table 3 presents the results of multiple logistic regressions, indicating that high-risk pregnancies could be twice the odds ratio of developing postpartum psychological distress (ß = 2.14, 95% CI 1.4-6.3, p= 0.036). The results also showed that high-risk pregnancy could almost double the odds ratio of developing postpartum psychological distress (ß = 2.14, 95% CI 1.4-6.3; p = 0.036). Moreover, it was noticed that pregnant women at high risk for mental health problems (scores above 23) had odds ratios of 22.5 times higher than those at low risk in terms of experiencing psychological distress in the postpartum period (ß= 22.46, 95% CI 6.74-42.68; p< 0.001). However, factors such as age and education level were not risk factors for postpartum psychological distress.

**Table 3:** The results of multiple logistic regression* for determinates of psychological distress of women in postpartum period

Variable	OR	CI 95%	P- value
High-risk pregnancy	2.14	1.06-4.30	0.036
Psychological risk > 23	22.46	6.68-74.42	<0.001
Age	1.04	0.98-1.11	0.148
Educational level	1	−	0.316
Primary school	1.41	0.49-4.06	0.517
University	0.75	0.29-1.92	0.556

*In this model, Psychological distress (GSI > 1) as a dependent variable, the psychosocial risk score ≥23, age, and education level as independent variables.

## Discussion

The present research was the first study comparing the severity of psychiatric symptoms and psychological distress in postpartum women with high-risk and low-risk pregnancies. The results confirmed the effect of high-risk pregnancy on the increase in the severity of psychiatric symptoms in the postpartum period.

This study also indicated that the mean scores of severe symptoms of obsession-compulsion, depression, anxiety, hostility, paranoid ideation, and psychoticism were significantly higher in postpartum women with high-risk pregnancies than in those with low-risk pregnancies. The frequency of the psychological distress index (GSI > 1) in postpartum women with high-risk pregnancies was twice as likely as that in postpartum women with low-risk pregnancies. To the best of our knowledge, no study has ever compared postpartum psychiatric problems (9 disorders) of pregnant women with low-risk and high-risk pregnancies; hence, we succeeded in comparing the means of the two groups in the present study to the ones in previous studies. However, few studies have reported one of the symptoms of 9 postpartum psychiatric disorders in either low-risk or high-risk groups. Consistent with the results, Chen et al. 26 concluded that the odds ratio of developing psychological disorders was higher in women with high-risk pregnancies. They also reported that postpartum depression in preeclampsia patients was twice (26.67%) as prevalent as in women with low-risk pregnancies. Another study reported that high-risk pregnancies were associated with an increased risk of postpartum psychological disorders. 27 One study also concluded that women with gestational diabetes had an increased risk of postpartum depression. 28 Contrary to the current report, another study did not report the severity of nine disorders and compare low-risk pregnancies. 29

Our results indicated that the frequency of risk factors for depression was approximately 1.5 times (59.8% vs. 39.8%) higher in women with high-risk pregnancies than in women with low-risk pregnancies. In line with the results, one study reported that women, who experienced high-risk pregnancy, had an increased risk of acute postpartum stress and depression in the postpartum period. 27 In that study, 16% of women with high-risk pregnancies needed hospitalization in the postpartum period due to psychiatric disorders. Among all patients admitted to the hospital,42, 6.30, 1.4, and 6.11% had anxiety disorders, depressive disorders, psychosis, and substance abuse, respectively. 30

The results of this study indicated that high-risk pregnancies and high scores of the risk factors for depression could increase the odds ratio of postpartum psychological distress twice and 22 times, respectively; however, age, education level, and numbers of pregnancies were not effective. Although no study was found on the risk factors for postpartum mental distress, some studies had examined the risk factors for postpartum psychological disorders. One study on 122 pregnant women with high-risk pregnancies reported that the scores of psychological distress in postpartum women were significantly correlated with their psychological distress scores during pregnancy (r = 0.67). Furthermore, the psychological distress of women with high-risk pregnancies increased significantly after delivery. 31 One study reported that low birth weight and lack of social support were the risk factors for psychological disorders in such women. 32 In one prospective study on 167 Chinese women with high-risk pregnancies, high scores of depression in the third trimester of pregnancy were considered a risk factor for postpartum depression. 33 In some studies with inconsistent results, women, who had their first pregnancy, were at a high risk of developing psychological disorders in the first month after delivery. 30

The results of the present study have many clinical implications in maternal care centers. The results suggest that all maternity healthcare providers, including obstetricians, midwives, and nurses should pay special attention to the identification and treatment of psychiatric problems in women with high-risk pregnancies, not only during pregnancy but also after delivery. In women with high-risk pregnancies, not only the risk of psychiatric problems is not eliminated after childbirth, but also it is more likely to increase. All healthcare providers of pregnant women should consider risk factors for psychiatric problems during pregnancy and after delivery. Identification and treatment of pregnant women with high-risk pregnancies, suffering from psychiatric problems, not only positively affect pregnancy outcomes, but also make the postpartum period less risky in terms of developing or exacerbating psychiatric problems.

The present study had several limitations. First, it was a case-control study, which cannot assess causation. Additionally, despite all the attempts made to control (by matching) important confounding variables, some effective factors like socioeconomic status were not controlled due to the patients' incorrect responses to income status. Second, in the study, high-risk pregnancy was not limited to one type of medical problem or risk factor, and each person was included in the study even with one factor. Therefore, there were 112 women with different causes of high-risk pregnancies. Owing to the small number of causes of high-risk pregnancy, it was impossible to conduct a supplementary analysis of subgroups and compare the severity of psychiatric symptoms in high-risk pregnancies with various causes. Thus, it is recommended that further studies be conducted as a prospective cohort on a population with a high number of high-risk pregnancies to compare the severity of psychiatric symptoms in two groups of women with low-risk and high-risk pregnancies from pregnancy to the postpartum period.

## Conclusion

The severity of numerous psychiatric symptoms and the psychological distress index were at higher levels in postpartum women with high-risk pregnancies than in those with low-risk pregnancies. Pregnant women with high-risk pregnancies or risk factors for psychological disorders were more likely to experience postpartum psychiatric distress. The study suggests that obstetricians should pay more attention to the identification and treatment of psychiatric symptoms in pregnant women with high-risk pregnancies, particularly those at high risk of developing psychological disorders, from pregnancy to the postpartum period.
